# Global value added and embodied carbon emission transfers driven by lithium battery trade

**DOI:** 10.1016/j.isci.2025.112320

**Published:** 2025-03-30

**Authors:** Meihui Jiang, Xiaoqing Hao, Bingyan Li, Wenlin Cai, Yanfang Zhang

**Affiliations:** 1 School of Management Science and Engineering, Nanjing University of Information Science & Technology, Nanjing 210044, China; 2 College of Economics and Management, Nanjing University of Aeronautics and Astronautics, Nanjing 211106, China

**Keywords:** Energy policy, Energy resources, Social sciences

## Abstract

The environmental consequences of the production and use of lithium batteries have been widely concerned in recent years. To uncover the environmental costs driven by lithium battery trade, this study traces value added and embodied carbon emission transfers using input-output model and network analysis. First, the results show the significant centralized trends occur as economies’ average exchange partners decreased by 3.0% (value added) and 7.7% (carbon emissions), while transfer volumes surged 6595% and 5953%. Second, Germany and the USA show the most significant value added and carbon emissions in imports, while China dominates exports. Third, imbalanced flows exist: European Union (EU) countries exhibit high valued but low-carbon imports, contrasting with the USA and UK’s low valued but carbon-intensive imports in 2021. Economies bearing notable environmental costs differ between imports (India) and exports (Mexico and Indonesia). These results inform lithium battery supply chain restructuring strategies by quantifying trade-driven environmental-economic linkages.

## Highlights

• Germany and the USA import high valued and carbon-intensive lithium batteries
• China exports lithium batteries with significant value added and carbon emissions
• India bears significant environmental costs in lithium battery imports
• Mexico and Indonesia bear notable environmental costs in lithium battery exports

## Graphical abstract

**Figure:**
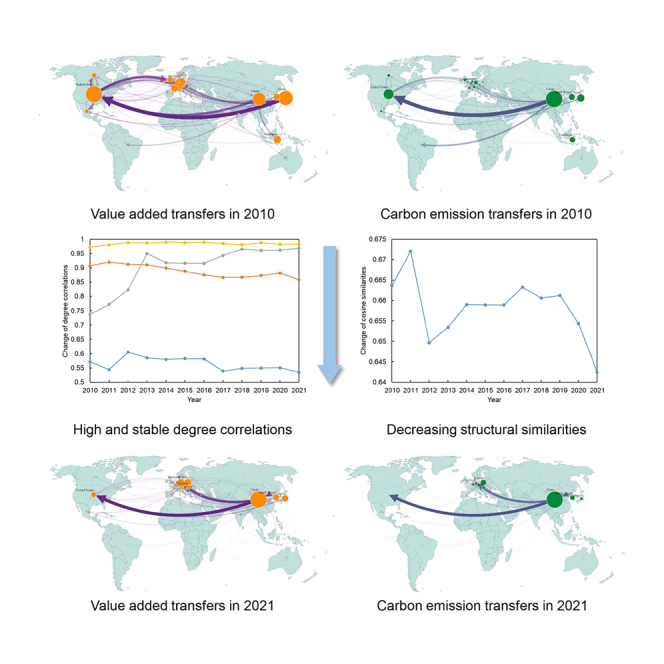

## Introduction

Research on new energy storage technologies has been driven by a series of environmental issues, including energy crisis, greenhouse effect, and air pollution, leading to the continuous development and commercialization of electrochemical energy storage batteries.^1^^,^^2^ The wide application of lithium battery in new energy vehicles has proved its value. The World Economic Forum predicts that the global battery demand will be 2,600 GWh in 2030.^3^ An assessment of carbon emissions from commercial electric vehicle batteries finds that cradle-to-gate greenhouse gas (GHG) emissions for a 24 kWh Ford Focus lithium battery are 140 kg CO2-eq per kWh.^4^ It means the global battery demand in 2030 will cause approximately 364 million tons CO2-eq of GHG emissions, which almost equals to 0.62% of total GHG emissions in 2023. Therefore, more and more countries are beginning to focus on the environmental consequences of the production and use of lithium batteries.^5^ For example, the European Union (EU) has issued new regulations concerning batteries and waste batteries (2023/1542). In the new regulations, power batteries exported to EU countries must hold a “battery passport” that contains the information of carbon footprint since 2027.^6^ Hence, for lithium battery exporters, it is important to build a higher valued and greener industrial chain of lithium battery.

In recent years, the economic and environmental issues on lithium battery have been hot topics due to the rapid development of new energy. In the economic issues on lithium battery, scholars widely focused on international trade patterns among countries of lithium battery. Scholars have studied the trade relationships of lithium resources in power battery between countries.^7^^,^^8^ Some studies have analyzed the evolution of trade patterns of lithium battery based on complex network.^9^^,^^10^ Due to the scarcity of lithium reserves, scholars also investigated the risk spreading along global lithium battery industry brought by the supply failure of lithium resources.^11^^,^^12^ In the environmental issues on lithium battery, scholars widely studied the environmental impacts of the production process of lithium battery, such as material requirements, energy consumption, greenhouse gas emissions, and water waste.^13^^,^^14^^,^^15^^,^^16^ Scholars analyzed the lithium flows in the economy based on the material flow analysis methods.^17^^,^^18^^,^^19^ Other scholars investigated the carbon footprint of lithium battery using the life cycle assessment approach.^20^ Some studies quantified the carbon footprint driven by lithium battery recycling and remanufacturing.^20^^,^^21^ The aforementioned research have deeply discussed the economic value brought by lithium battery trade and the carbon footprint in lithium battery production. However, the studies on the economic value and the carbon footprint of lithium battery production and use mostly are independent. Few scholars provided the integrated comparison analysis on value and carbon emission flows along the lithium battery industrial chain, which makes the environmental costs of economic profits of lithium battery production and sales in different countries unclear. For lithium battery exporters, increasing value added and decreasing carbon emissions in lithium battery exports can yield high economic benefits while avoiding carbon emission limitations from importers. It is also beneficial for controlling global carbon emissions. Hence, it is necessary to quantify the value added and carbon emission transfers among countries driven by lithium battery trade.

As for the method, the input-output analysis provides a useful tool for building the integrated framework to quantify the value added and carbon emissions driven by lithium battery trade. There have been many studies that applied the input-output analysis to account carbon emissions embodied in the trade among countries.^22^^,^^23^^,^^24^ Scholars also proposed some methods to decompose value added from gross trade value based on the input-output analysis, including the HIY (Hummels, Ishii, and Yi) method,^25^ the KWW (Koopman, Wang, and Wei) method^26^ and the hypothesis extraction method (HEM).^27^ Hence, we can quantify both of value added and carbon emission transfers among countries by applying the input-output analysis. However, one of the limitations of the input-output table is that the specific products are usually integrated into sectors. Most of the current datasets of global input-output table have not included specific data on lithium battery. Hence, it is difficult to extract lithium battery data from current global input-output table. How to combine the global input-output table with international trade data of lithium battery is the key to accounting value added and embodied carbon emissions driven by lithium battery trade. To solve this problem, this paper applies the idea of the HEM in input-output analysis. The HEM refers to measure the impact of a certain economic connection on economic variables by eliminating such economic connection from the economic system and estimating the change rate of economic variables before and after the elimination. It has been widely used to account value added, carbon footprint, and material footprint.^28^^,^^29^^,^^30^ By assuming that there are no trade data of lithium battery in the original global input-output table, the value added and carbon emissions driven by lithium battery trade can be quantified by the differences before and after adding trade data of lithium battery into the global input-output table. Furthermore, due to the multi-agent and multi-relationship complicated structure, this paper applies multiplex network to describe the characteristics of the value added and carbon emission flows among countries. Multiplex network is expanded from complex network, which has been widely used to study the complicated economic structure and social relationship structure with multiple types of relationships.^31^^,^^32^^,^^33^^,^^34^

In this paper, our main purpose is to uncover the environmental costs in different economies driven by lithium battery trade. Different from the existing research, this paper proposes an integrated model framework to quantify the value added and carbon emissions transfers among economies driven by lithium battery trade by combining the input-output analysis, the HEM and multiplex network. First, we account value added and embodied carbon emission flows among economies driven by lithium battery trade. Second, we construct a two-layer lithium-battery-trade-driven value-emission flow network (LBTVEFN) that contains lithium-battery-trade-driven value added flow network (LBTVAFN) and lithium-battery-trade-driven carbon emission flow network (LBTCEFN) and analyze the structure features of value added and embodied carbon emission transfers among economies. Third, we deeply analyze the relationships between global transfers of value added and embodied carbon emissions.

## Results

### Overall network structure characteristics of the LBTVEFN

#### Overall structural characteristics of the LBTVAFN

**Figure 1:**
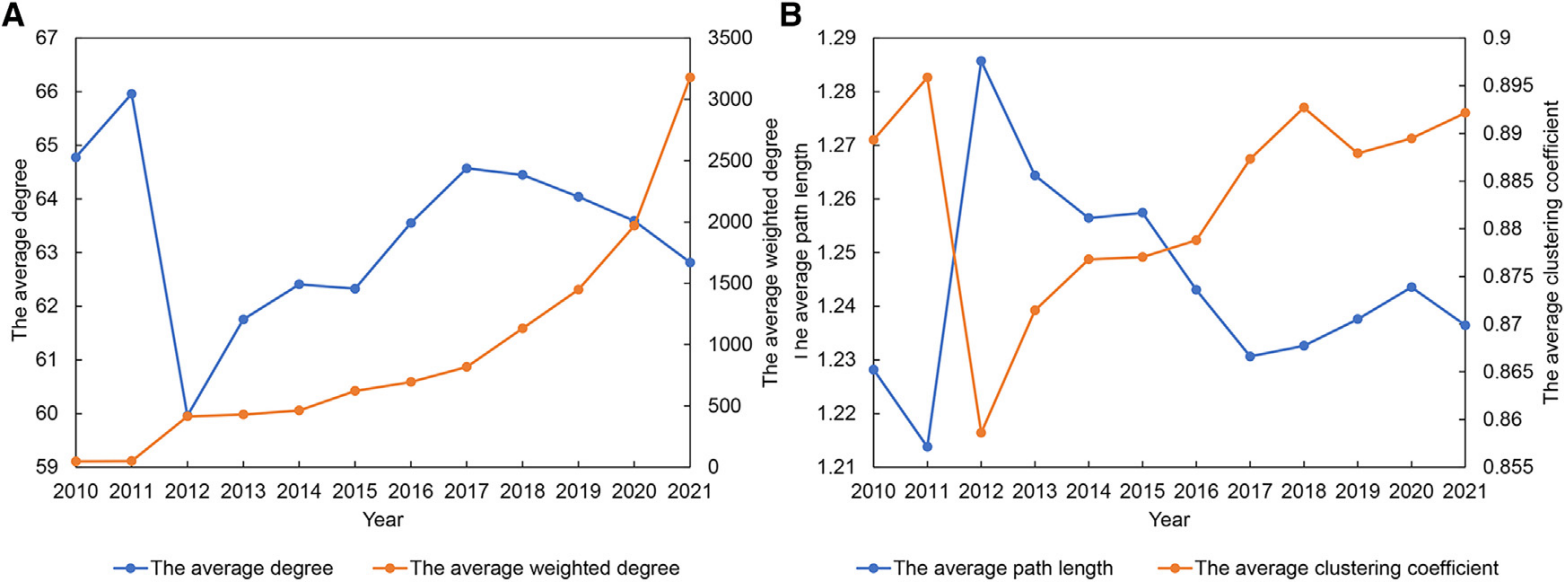
Structural characteristics of the LBTVAFN in 2010–2021 (A) The average degree and the average weighted degree of the LBTVAFN in 2010–2021. (B) The average path length and the average clustering coefficient of the LBTVAFN in 2010–2021.

As shown in Figure 1A, there is a significant fluctuation in the trend of the average degree of the LBTVAFN in 2010–2021. In 2010–2012, the change of average degree is very dramatic. The average degree of the LBTVAFN significantly decreases in 2011–2012 after the short increase in 2010–2011. During the period between 2012 and 2017, the average degree of the LBTVAFN significantly increases, and then significantly decreases from 2017 to 2021. The average weighted degree keeps increasing in 2010–2021. Generally, the average degree of the LBTVAFN decreases by 3.0% and the average weighted degree increases by 6595% in 2010–2021. It indicates that although the average exchange partners of economies in value added transfers decrease, the average transfer volumes of economies significantly increase. It reflects that value added transfers driven by lithium battery trade may have a centralized trend. As shown in Figure 1B, the average path length and the clustering coefficient of the LBTVAFN also change dramatically in 2010–2012. The clustering coefficient of the LBTVAFN significantly decreases in 2011–2012 following a brief increase in 2010–2011, while the change of average path length exhibits the opposite trend. It indicates the connectivity of economies in the LBTVAFN decreases. In 2012–2021, Figure 2B shows that there is an increased trend in the average path lengths and a decreased trend in the clustering coefficients of the LBTVAFN. It indicates that the connectivity of economies in the LBTVAFN increases in 2010–2021. It reflects the network density of the LBTVAFN increases in recent years.

**Figure 2:**
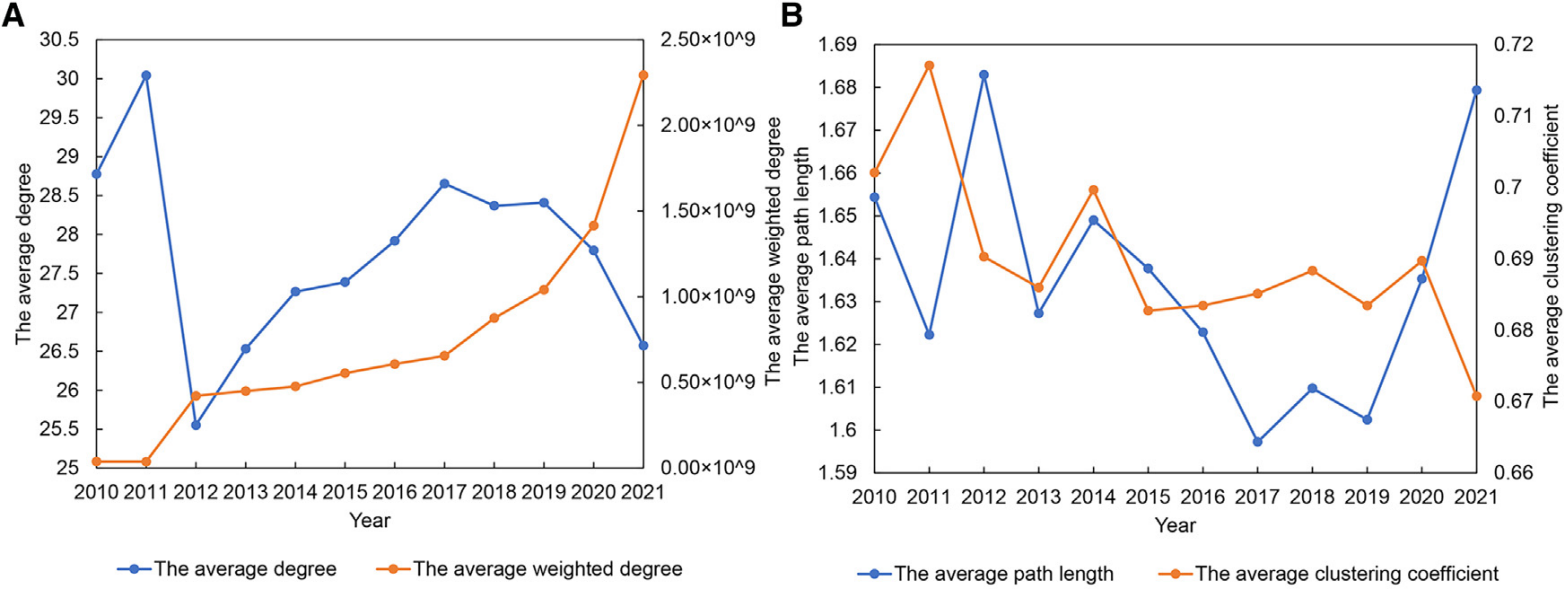
Structural characteristics of the LBTCEFN in 2010–2021 (A) The average degree and the average weighted degree of the LBTCEFN in 2010–2021. (B) The average path length and the average clustering coefficient of the LBTCEFN in 2010–2021.

#### Overall structural characteristics of the LBTCEFN

As shown in Figure 2A, the change in the average degree and the average weighted degree in the LBTCEFN is much consistent with that in the LBTVAFN. Generally, the average degree of the LBTCEFN decreases by 7.7% and the average weighted degree increases by 5953% in 2010–2021. It indicates that although the average exchange partners of economies in embodied carbon emission transfers decrease, the average transfer volumes of economies significantly increase. It reflects that embodied carbon emission transfers driven by lithium battery trade may also have a centralized trend. According to Figure 2B, the average path lengths of the LBTCEFN firstly decreases in 2012–2017, then increases in 2017–2021. The average clustering coefficients of the LBTCEFN keeps decreasing during the period from 2010 to 2021. It reflects that the network density of the LBTCEFN decreases in recent years.

### The roles of economies in the LBTVEFN

#### Economies’ roles in the LBTVAFN

**Figure 3:**
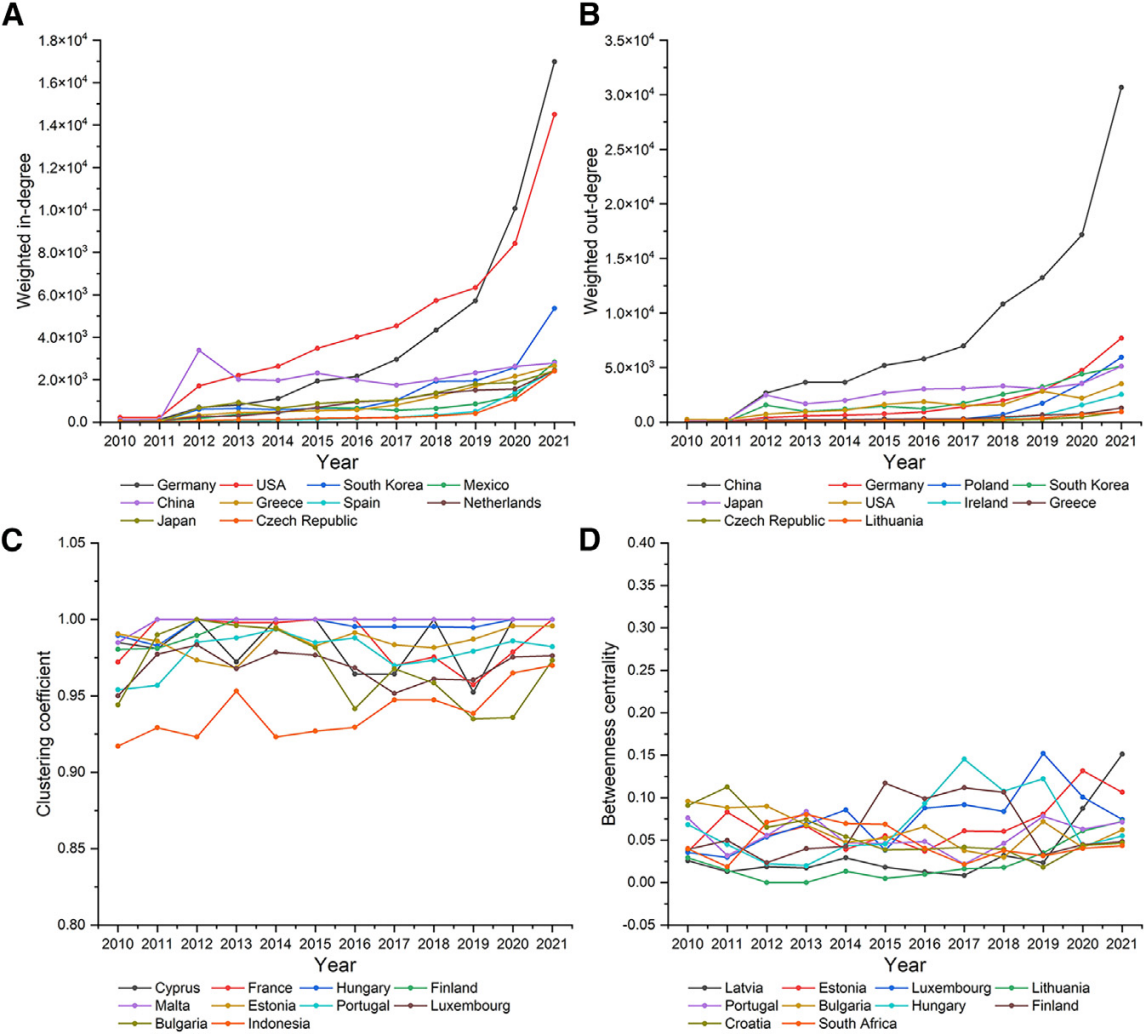
The roles of the top 10 economies in the LBTVAFN in 2010–2021 (A) Weighted in-degree of the top 10 economies in the LBTVAFN in 2010–2021. (B) Weighted out-degree of the top 10 economies in the LBTVAFN in 2010–2021. (C) Clustering coefficient of the top 10 economies in the LBTVAFN in 2010–2021. (D) Betweenness centrality of the top 10 economies in the LBTVAFN in 2010–2021.

As shown in Figure 3A, there is an increasing trend in the weighted in-degree of the selected economies in 2010–2021. It indicates that the value added in the imports related to lithium batteries in the selected economies increase in 2010–2021. It may be caused by the increasing demands of lithium batteries in these years. The important economies with high value added in the imports related to lithium batteries include Germany and the USA. The value added in the imports in these two economies have accounted by 40.4% of total value added in 2021. As shown in Figure 3B, there is an increasing trend in the weighted out-degree of the selected economies in 2010–2021. It indicates that the value added in the exports related to lithium batteries in the selected economies increase in 2010–2021. It may be caused by the growth of production and export of lithium batteries in these years. The most important economy with high value added in the exports related to lithium batteries is China. The value added in the exports in China have accounted by 41.9% of total value added in 2021. According to the trade data, Germany and the USA are the most important importers and China is the most important exporter of lithium batteries (Table S1). Hence, the transfers of value added may be mainly influenced by lithium battery trade. As shown in Figures 3C and 3D, most of the economies with high clustering coefficient and betweenness centrality are EU countries. It means Europe is the center of value added exchanges in lithium battery trade.

#### Economies’ roles in the LBTCEFN

**Figure 4:**
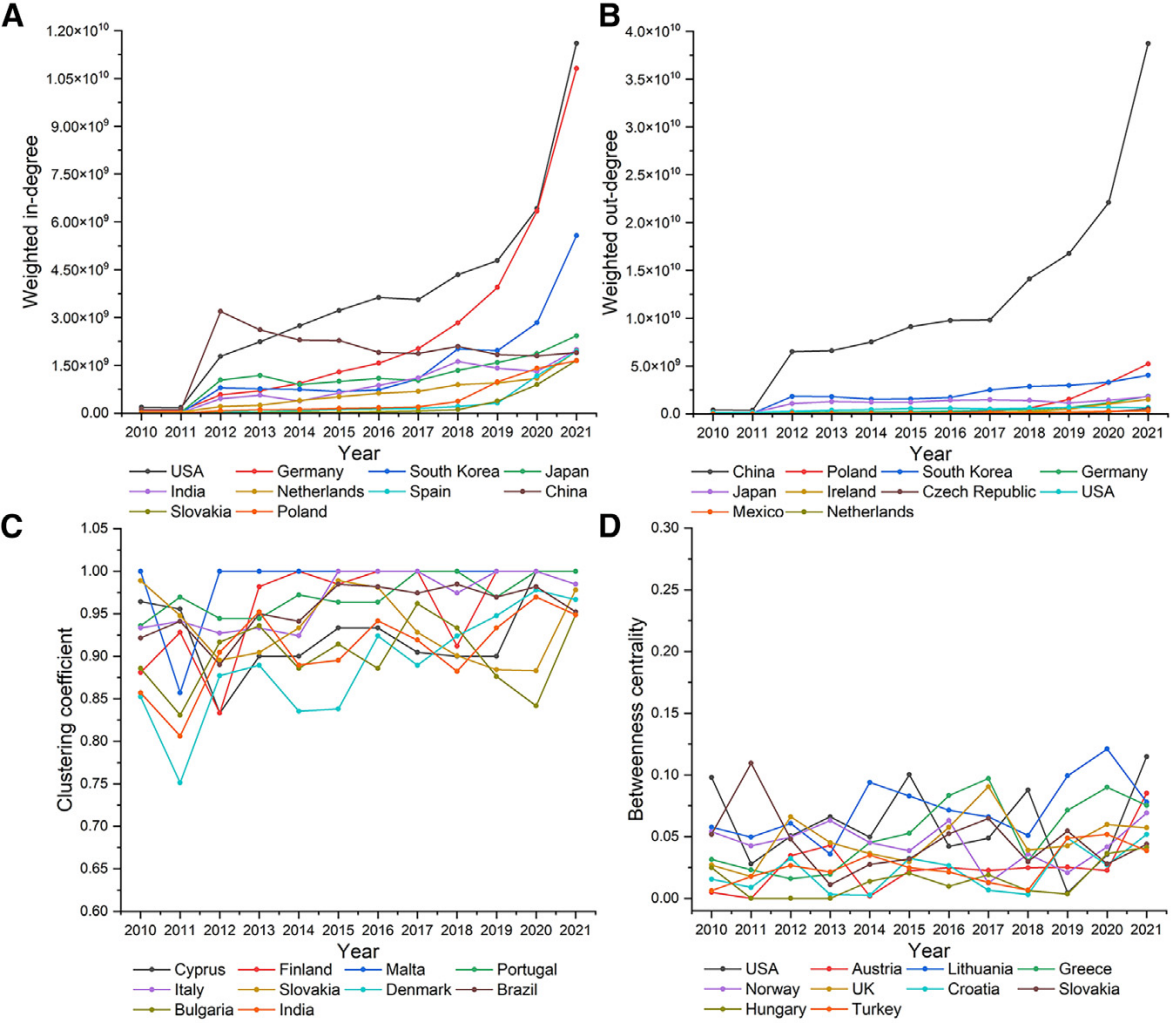
The roles of the top 10 economies in the LBTCEFN in 2010–2021 (A) Weighted in-degree of the top 10 economies in the LBTCEFN in 2010–2021. (B) Weighted out-degree of the top 10 economies in the LBTCEFN in 2010–2021. (C) Clustering coefficient of the top 10 economies in the LBTCEFN in 2010–2021. (D) Betweenness centrality of the top 10 economies in the LBTCEFN in 2010–2021.

As shown in Figure 4A, there is an increasing trend in the weighted in-degree of the selected economies in 2010–2021. It indicates that the embodied carbon emissions in the imports related to lithium batteries increases in 2010–2021. The important economies in the LBTCEFN with high imported embodied carbon emissions in lithium battery trade include Germany and the USA. The embodied carbon emissions in the imports in these two economies have accounted by 39.9% of total embodied carbon emissions in 2021. As shown in Figure 4B, there is an increasing trend in the weighted out-degree of the selected economies in 2010–2021. It indicates that the embodied carbon emissions in the exports related to lithium batteries increases in 2010–2021. The most important economy in the LBTCEFN with high exported embodied carbon emissions in lithium battery trade is China. The embodied carbon emissions in the exports in China have accounted by 68.9% of total embodied carbon emissions in 2021. As we mentioned before, Germany and the USA are the most important importers and China is the most important exporter of lithium batteries (Table S1). Hence, the transfers of embodied carbon emissions may also be mainly influenced by lithium battery trade. As shown in Figures 4C and 4D, most of the economies with high clustering coefficient and betweenness centrality are European economies. It means Europe is the center of carbon emission exchanges in lithium battery trade.

Overall, the weighted in-degree and weighted out-degree of economies in the LBTCEFN are consistent with that in the LBTVAFN, which means that the imports and exports related to lithium batteries in most economies are usually high valued and carbon-intensive, such as Germany, the USA, and China. However, there are also some economies playing different roles in the LBTVAFN and the LBTCEFN. The economic costs driven by lithium battery trade in India, Slovakia, and Poland are not very high but embodied carbon emissions are very significant, which means that the environmental costs of imports related to lithium batteries in these three economies are relatively high. The value added in the imports related to lithium batteries in Mexico, Greece, and Czech Republic are high but embodied carbon emissions are not very significant, which means that the environmental costs of the imports related to lithium batteries in these three economies are relatively low. The value added in the exports related to lithium batteries in Mexico and Indonesia are not very high but embodied carbon emissions are very significant, which means the environmental costs of the exports related to lithium batteries in these two economies are relatively high. The value added in the exports related to lithium batteries in Greece and Lithuania are high but embodied carbon emissions are not very significant, which means the environmental costs of the exports related to lithium batteries in these two economies are relatively low. The value added in the exports related to lithium batteries in Mexico and Indonesia are not very high but embodied carbon emissions are very significant, which means the environmental costs of exports related to lithium batteries in these two economies are relatively high.

### The relationships between the LBTVAFN and the LBTCEFN

#### Comparisons between weighted degree of economies in the LBTVAFN and the LBTCEFN

**Figure 5:**
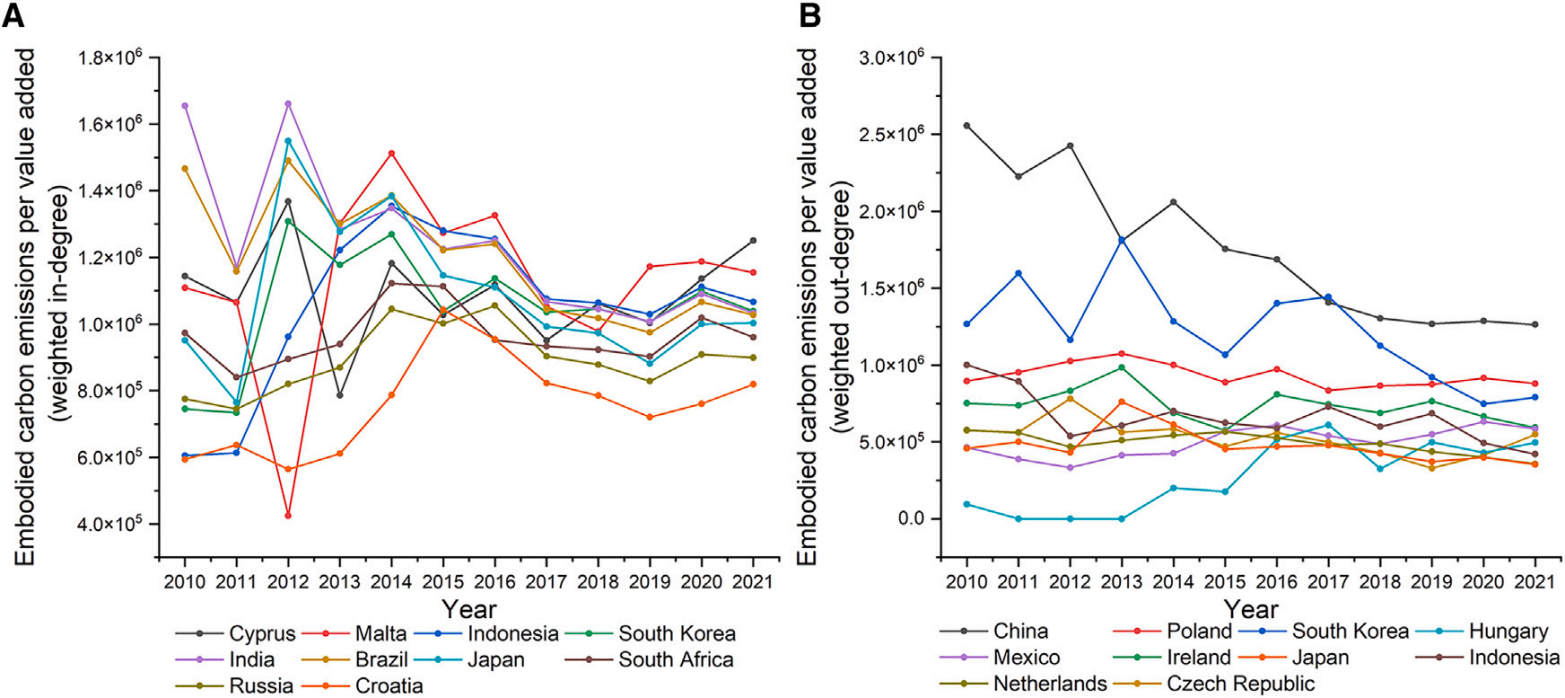
Embodied carbon emissions per value added of the top 10 economies in 2010–2021 (A) The ratio of weighted in-degrees of economies in the LBTCEFN and the LBTVAFN. (B) The ratio of weighted out-degrees of economies in the LBTCEFN and the LBTVAFN.

As we mentioned before, we find that there are some economies with different environmental costs in imports and exports related to lithium batteries. To further detect the economies with significant environmental costs driven by lithium battery trade, we calculate the ratio of weighted in-degrees of economies in the LBTCEFN and the LBTVAFN as embodied carbon emissions per value added in the imports driven by lithium battery trade. As shown in Figure 5A, the economies with significant embodied carbon emissions per value added in the imports includes Cyprus, Malta, Indonesia, South Korea, and India. We find embodied carbon emissions per value added in the imports of some economies slightly decrease in recent years, such as India, Brazil, and Japan. It indicates that these economies’ environmental costs of lithium battery imports decrease, which means the import sources of these economies are more and more environment-friendly. There are some economies whose embodied carbon emissions per value added in the imports slightly increased in recent years, such as Cyprus, Indonesia, and Croatia. It indicates that these economies’ environmental costs of lithium battery imports increased, which means the import sources of these economies are more and more environment-unfriendly.

We also calculate the ratio of weighted out-degrees of economies in the LBTCEFN and the LBTVAFN as embodied carbon emissions per value added in the exports driven by lithium battery trade. As shown in Figure 5B, the economies with significant embodied carbon emissions per value added in the exports includes China, Poland, South Korea, Ireland, Czech Republic, Mexico, and Indonesia. We find that embodied carbon emissions per value added in the exports of some economies significantly decreased in recent years, such as China and South Korea. It indicates that these economies’ environmental costs of lithium battery exports decreased, which means the exports related to lithium batteries of these economies are becoming more and more environment-friendly. There are some economies whose embodied carbon emissions per value added in the exports slightly increased in recent years, such as Ireland and Hungary. It indicates that these economies’ environmental costs of lithium battery exports increased, which means the exports related to lithium batteries of these economies are becoming more and more environment-unfriendly.

#### Structural similarity between the LBTVAFN and the LBTCEFN

**Figure 6:**
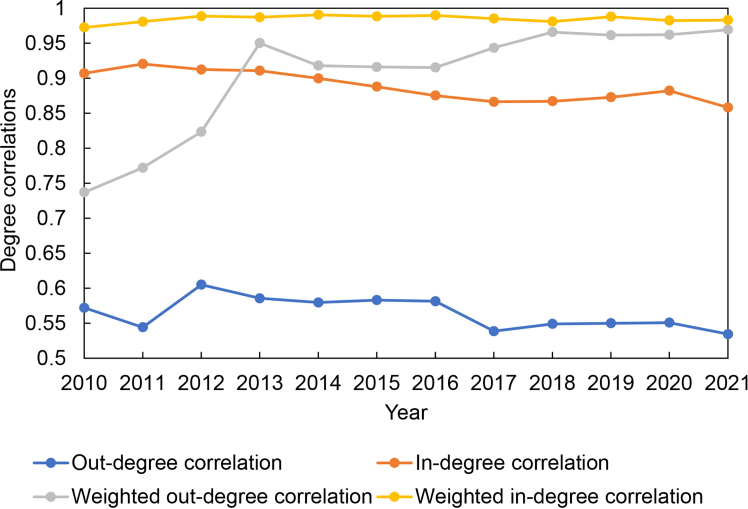
Degree correlations between the LBTVAFN and the LBTCEFN in 2010–2021

As shown in Figure 6, the weighted in-degree correlation coefficients between the LBTVAFN and the LBTCEFN are high and stable in 2010–2021. It means the inflow volume of value added and embodied carbon emissions of economies is commonly consistent. There is a slightly decreased trend in the in-degree correlation coefficients between the LBTVAFN and the LBTCEFN in 2010–2021. It means the similarity between the number of source partners of value added and embodied carbon emissions of economies slightly decreased. It reflects that although economies commonly import high valued and carbon-intensive goods driven by lithium battery trade, the import sources of the related goods may be different. The weighted out-degree correlation coefficients between the LBTVAFN and the LBTCEFN have a significantly increased trend in 2010–2021. It indicates that the outflow volume of value added and embodied carbon emissions of economies is becoming more and more similar. However, there is a slightly decreased trend in out-degree correlation coefficients between the LBTVAFN and the LBTCEFN in 2010–2021. It means the number of target partners of value added and embodied carbon emissions of economies is different from each other. Hence, changes of degree correlations between the LBTVAFN and the LBTCEFN indicate that there may be some good flows between economies whose embodied carbon emissions and value added are not balanced.

**Figure 7:**
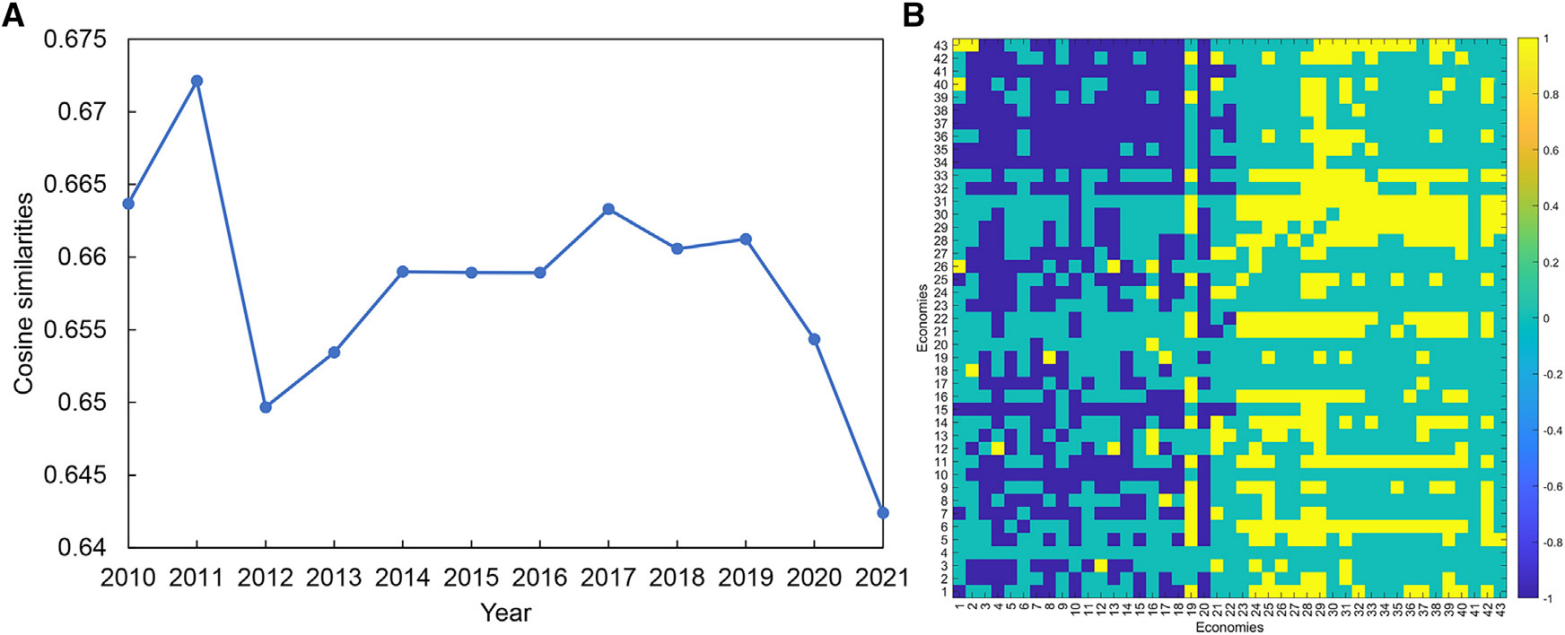
Structural similarities between the LBTVAFN and the LBTCEFN (A) Cosine similarity between the LBTVAFN and the LBTCEFN in 2010–2021. (B) Difference matrix between the LBTVAFN and the LBTCEFN in 2021.

As shown in Figure 7A, the cosine similarities between the LBTVAFN and the LBTCEFN significantly decreased during the period from 2010 to 2021. It indicates that the edge pairs in the LBTVAFN and the LBTCEFN are becoming more and more different. It further proves that there exist some good flows between economies whose embodied carbon emissions and value added are not equal. Figure 7B is the difference matrix in 2021 obtained by $A^{LBTCEFN}-A^{LBTVAFN}$, where $A^{LBTCEFN}$ denotes the adjacent matrix of the LBTCEFN and $A^{LBTVAFN}$ denotes the adjacent matrix of the LBTVAFN. We can see there were three kinds of edge pair differences between the LBTVAFN and the LBTCEFN. Most edge pairs exist or do not exist both in the LBTVAFN and the LBTCEFN. Some edge pairs exist in the LBTVAFN but not in the LBTCEFN. All these edges are from other economies to EU countries. It indicates that most EU countries intend to import high valued but low-carbon goods related to lithium batteries. Other edge pairs exist in the LBTCEFN but not in the LBTVAFN. Most of these edges are from other economies to the USA, the UK, and China. It indicates that these economies intend to import low valued but carbon-intensive goods related to lithium batteries.

## Discussion

The major purpose of this paper is to uncover the environmental costs in different economies driven by lithium battery trade to help lithium battery exporters build higher valued and greener industrial chains. Comparing with the previous studies, our main contribution is that we propose an integrated model framework to quantify the value added and carbon emissions transfers among economies driven by lithium battery trade by combining the input-output analysis, the HEM and multiplex network, which can be also applied to the related study of other specific commodities. The main findings are as follows.

First, the results show that there are significant centralized trends of value added and embodied carbon emission transfers driven by lithium battery trade. The average degree of the LBTVAFN and the LBTCEFN decreases by 3.0% and 7.7% in 2010–2021, while the average weighted degree of the LBTVAFN and the LBTCEFN increases by 6595% and 5953%. It indicates that although the average exchange partners of economies in value added and embodied carbon emission transfers driven by lithium battery trade decrease, the average transfer volumes of economies significantly increase. The changes of the average path length and average clustering coefficient show that the network density of the LBTVAFN increases while the network density of the LBTCEFN deceases. It indicates that compared with embodied carbon emission flows, the transfers of value added among economies are more frequent.

Second, the results show that there are some economies which play important roles in the global transfers of value added and embodied carbon emissions driven by lithium battery trade. The roles of most economies in value added and embodied carbon emission transfers are consistent. The value added and embodied carbon emissions in the imports related to lithium batteries in Germany and the USA are most significant, which account by 40.4% and 39.9% of total value added and embodied carbon emissions in 2021. The value added and embodied carbon emissions in the exports related to lithium batteries in China are most significant, which account by 41.9% and 68.9% of total value added and embodied carbon emissions in 2021. Europe is an important trade center of global transfers of value added and embodied carbon emissions driven by lithium battery trade. However, there were also some economies whose roles in value added and embodied carbon emission transfers are not consistent. The economic costs driven by lithium battery imports in India, Slovakia, and Poland are not very high but embodied carbon emissions are very significant. The economic costs driven by lithium battery imports in Mexico, Greece, and Czech Republic are high but embodied carbon emissions are not very significant. The economic incomes driven by lithium battery exports in Mexico and Indonesia are not very high but embodied carbon emissions are very significant. The economic incomes driven by lithium battery exports in Greece and Lithuania are high but embodied carbon emissions are not very significant.

Third, the results show that there are some good flows driven by lithium battery trade among economies whose value added and embodied carbon emissions are not balanced. Comparisons between weighted degrees of economies in the LBTVAFN and the LBTCEFN show that there are both increased and decreased trends in embodied carbon emissions per value added in the imports and the exports driven by lithium battery trade. It indicates that the differences among economies in environmental costs of the lithium battery imports and the exports are narrowing. On the import side, the economies with significant environment costs include Cyprus, Malta, Indonesia, South Korea, and India. One the export side, the economies with significant environment costs include China, Poland, South Korea, Ireland, Czech Republic, Mexico, and Indonesia. The results of network structural similarities further show that although there is high consistency between the flow trajectory of value added and embodied carbon emissions, the consistency is decreasing. EU countries intend to import high valued but low-carbon goods related to lithium batteries, while the USA, the UK, and China intend to import low valued but carbon-intensive goods in 2021.

The results suggest that there are significantly consistent relationships between value added and embodied carbon emission transfers driven by lithium battery trade. It indicates that the current production of lithium batteries is still highly polluted and highly valued. For important lithium battery exporters such as China, the investment in technology research and development should be strengthened to reduce carbon emissions in the production process of the products related to lithium batteries. For important lithium battery importers such as Germany and the USA, the import sources and structures of the products related to lithium batteries should be adjusted to reduce the impacts on global carbon emissions brought by the consumption of lithium batteries.

The results also suggest that there are some good flows driven by lithium battery trade among economies whose value added and embodied carbon emissions are not balanced. It indicates there are some trade flows driven by lithium battery whose economic benefits and environmental costs are not equal. For some economies with high environment costs in the imports related to lithium batteries such as India, their import sources should be significantly adjusted. The import sources of EU countries can be good references. Some economies with high incomes and low embodied carbon emissions in the exports related to lithium batteries, such as Greece and Lithuania, should be further encouraged to participate in the global industrial chain of lithium batteries. For some economies with low incomes and high embodied carbon emissions in the exports related to lithium batteries such as Mexico and Indonesia, industrial transformation may be a better option considering the strict limitation of carbon footprint in the near future.

### Limitations of the study

Due to the limitation of the classification of sectors in the current global input-output tables, there is no single sector producing lithium batteries. In this paper, we assume the sector that produces lithium battery is sector 120 (Electrical machinery and apparatus n.e.c.) according to the definition of sectors in EXIOBASE. It may limit the accuracy of the results. We will improve this in the future by dividing lithium battery processing sector from the existing sectors.

## Resources availability

### Lead contact

Further information and requests for resources should be directed to and will be fulfilled by the lead contact, Xiaoqing Hao (hxqrenwen308@163.com).

### Materials availability

This study did not generate new unique reagents.

### Data and code availability

• All data reported in this paper will be shared by the lead contact upon request.
• This paper does not report original code.
• Any additional information required to reanalyze the data reported in this paper is available from the lead contact upon request.

## Acknowledgments

This research is supported by grants from the 10.13039/501100001809National Natural Science Foundation of China (grant no. 42301342, 42201330, 71991481, 71991485, 71991480, 72104107, and 72474102).

## Author contributions

Conceptualization, M.J. and X.H.; methodology, M.J. and X.H.; investigation, M.J.; formal analysis, M.J.; resources, M.J.; writing – original draft, M.J.; writing – review & editing, B.L. and W.C.; visualization, M.J.; supervision, X.H. and Y.Z.; funding acquisition, X.H. and Y.Z. All authors have agreed to submit the manuscript, read, and approved the final draft and take full responsibility of its content, including the accuracy of the data and the fidelity of the trial to the registered protocol and its statistical analysis.

## Declaration of interests

The authors declare no competing interests.

## STAR★Methods

### Key resources table

**Table:** 

REAGENT or RESOURCES	SOURCE	IDENTIFIER
**Deposited data**		

International trade data of lithium battery, including lithium-ion electric accumulators (HS code: 85076) and lithium cells and batteries (HS code: 85065).	UN COMTRADE database	https://comtradeplus.un.org/, Table S1
Global input-output tables in 2010-2021.	EXIOBASE database	https://www.exiobase.eu/, Table S2

**Software and algorithms**		

Matlab R2021b	Commercially Available Software	N/A
R studio 2024.04.0	Commercially Available Software	N/A
Origin 2022	Commercially Available Software	N/A
Scimago Graphica Beta 1.0.46	Commercially Available Software	N/A

Note: The unit of international trade data of lithium battery is the US dollar. The unit of the EXIOBASE input-output tables is the million euro. In this paper, we use the exchange rate in 2010-2021 to convert the unit of international trade data to million euro. The global input-output tables are provided at the current price. To eliminate the influence of price changes when calculating embodied carbon emissions, we use the Gross Domestic Product (GDP) deflator (constant 2015 euro) to transform the current price to constant price from 2015.

### Method details

#### Lithium-battery-trade-driven global value chain accounting

In this paper, we apply the improved KWW method (the WWZ (Wang, Wei & Zhu) method), to decompose gross trade into value added. The WWZ method was based on multi-regional input-output analysis. The structure of a multi-regional input-output table with 3 economies and 2 sectors is shown in Figure S1. The yellow part is the intermediate matrix Z, which is calculated by $A\hat{x}$. A is the direct consumption coefficient matrix, and $\hat{x}$ is the diagonal matrix of total output. $Z^{rs}$ is sectoral intermediate input/output from economy s to economy r (similar with $Z^{rr},Z^{tt},Z^{rt},Z^{tr},Z^{sr},Z^{ts}\;\text{and}\;Z^{ss}$). The blue part is the final consumption matrix Y. $y^{rs}$ is the vector of final consumption exported to economy r from sectors in economy s (similar with $y^{rr},y^{tt},y^{rt},y^{tr},y^{sr},y^{ts}\;\text{and}\;y^{ss}$). The green parts are the total output vector and total input vector. $x^{r}$ is the vector of sectoral total output in economy r (similar with $x^{s},x^{t}$). Its transpose ${\left(x^{r}\right)}^{\prime }$ is the vector of sectoral total input in economy r. The red part is the vector of value added. ${\text{va}}^{r}$ is the vector of sectoral value added in economy r (similar with ${\text{va}}^{s},{\text{va}}^{t}$).

The WWZ method aims to decompose exports between economies into 4 parts which contain 16 components in total:

(Equation 1) $$e^{sr}=Z^{sr}+y^{sr}=\text{dva}+\text{rva}+\text{fva}+\text{pdc}$$

dva is domestic value added absorbed abroad. fva is foreign value added. rva is value added first exported but eventually returned home. pdc is pure double counted terms. Except pure double counted terms pdc, other 3 parts of exports are calculated as:

(Equation 2) $$\text{dva}={\left(v^{s}B^{ss}\right)}^{\prime }\#y^{sr}+{\left(v^{s}L^{ss}\right)}^{\prime }\#A^{sr}B^{rr}y^{rr}+{\left(v^{s}L^{ss}\right)}^{\prime }\#A^{sr}B^{rt}y^{tt}+{\left(v^{s}L^{ss}\right)}^{\prime }\#A^{sr}B^{rr}y^{rt}+{\left(v^{s}L^{ss}\right)}^{\prime }\#A^{sr}B^{rt}y^{tr}$$

(Equation 3) $$\text{rva}={\left(v^{s}L^{ss}\right)}^{\prime }\#A^{sr}B^{rr}y^{rs}+{\left(v^{s}L^{ss}\right)}^{\prime }\#A^{sr}B^{rt}y^{ts}+{\left(v^{s}L^{ss}\right)}^{\prime }\#A^{sr}B^{rt}y^{ss}$$

(Equation 4) $$\text{fva}={\left(v^{r}B^{rs}\right)}^{\prime }\#y^{sr}+{\left(v^{r}B^{rs}\right)}^{\prime }\#A^{sr}L^{rr}y^{rr}+{\left(v^{t}B^{ts}\right)}^{\prime }\#y^{sr}+{\left(v^{t}B^{ts}\right)}^{\prime }\#A^{sr}L^{rr}y^{rr}$$

A is direct consumption coefficient matrix, and $A^{sr}$ is the block in A which contains the intermediate inputs from economy s required by economy r for per unit of total outputs. B is Leontief inverse matrix which is calculated by ${\left(I-A\right)}^{-1}$. $B^{rs}$ is the block in B which contains the total amount of direct and indirect intermediate inputs from economy r required by economy s for per unit of final consumption (similar with $B^{ss}$, $B^{rr}$, $B^{rt}$ and $B^{ts}$). $L^{ss}$ is the Leontief inverse matrix in economy s which is calculated by ${\left(I-A^{ss}\right)}^{-1}$ (similar with $L^{rr}$). $v^{r}$ is the vector of value added coefficient, which is obtain by ${\text{va}}^{r}{\left(x^{r}\right)}^{-1}$ (similar with $v^{s},v^{t}$).

Based on the idea of the HEM, we assume that there are no trade data of lithium battery in final consumption in the original global input-output table (Note: In this paper, we focus on lithium battery industrial chain. Hence, we treat all trading lithium batteries as final consumption products). Therefore, the global value chain accounted based on the original global input-output table won’t contain the value added brought by lithium battery trade. Then, we add trade data of lithium battery into final consumption of one sector which produces lithium battery, then use adjusted global input-output table to account global value chain which contains the value added brought by lithium battery trade. The difference between the two results of global value chains is the global value chain purely driven by lithium battery trade. Assuming that battery is produced by sector ϕ, the final consumption of the sector ϕ should be adjusted as:

(Equation 5) $${\bar{y}}_{\phi }^{sr}=y_{\phi }^{sr}+t^{sr}$$

$y_{\phi }^{sr}$ is the original final consumption of sector ϕ in economy s exported to economy r. $t^{sr}$ is the export data of lithium battery from economy s to economy r. Let ${\bar{y}}^{rs}$ denote the adjusted vector of final consumption exported to economy r from sectors in economy s which includes the export data of lithium battery from economy s to economy r (similar with ${\bar{y}}^{rr},{\bar{y}}^{tt},{\bar{y}}^{rt},{\bar{y}}^{tr},{\bar{y}}^{sr},{\bar{y}}^{ts}\;\text{and}\;{\bar{y}}^{ss}$). Expand to all economies, we can obtain an adjusted final consumption matrix including the lithium battery trade $\bar{Y}$. Due to the change of final consumption, the total output vector x will also change ($x_{\phi }^{s^{\prime }}=x_{\phi }^{s}+t^{sr}$). Let $\bar{x}$ be the adjusted total output, the adjusted intermediate matrix should be $\bar{Z}=A\hat{\bar{x}}$, then the value added vector should be $\bar{\text{va}}={\bar{x}}^{\prime }-\tilde{\left(\bar{Z}\right)}$ ($\tilde{\left(\bar{Z}\right)}$ denotes the intermediate input vector). The adjusted value added coefficient vector $\bar{v}$ should be calculated by $\bar{v}{\left(\bar{x}\right)}^{-1}$. Therefore, the adjusted value added parts in exports from economy s to economy r which include lithium battery should be:

(Equation 6) $$\bar{\text{dva}}={\left({\bar{v}}^{s}B^{ss}\right)}^{\prime }\#{\bar{y}}^{sr}+{\left({\bar{v}}^{s}L^{ss}\right)}^{\prime }\#A^{sr}B^{rr}{\bar{y}}^{rr}+{\left({\bar{v}}^{s}L^{ss}\right)}^{\prime }\#A^{sr}B^{rt}{\bar{y}}^{tt}+{\left({\bar{v}}^{s}L^{ss}\right)}^{\prime }\#A^{sr}B^{rr}{\bar{y}}^{rt}+{\left({\bar{v}}^{s}L^{ss}\right)}^{\prime }\#A^{sr}B^{rt}{\bar{y}}^{tr}$$

(Equation 7) $$\bar{\text{rva}}={\left({\bar{v}}^{s}L^{ss}\right)}^{\prime }\#A^{sr}B^{rr}{\bar{y}}^{rs}+{\left({\bar{v}}^{s}L^{ss}\right)}^{\prime }\#A^{sr}B^{rt}{\bar{y}}^{ts}+{\left({\bar{v}}^{s}L^{ss}\right)}^{\prime }\#A^{sr}B^{rt}{\bar{y}}^{ss}$$

(Equation 8) $$\bar{\text{fva}}={\left({\bar{v}}^{r}B^{rs}\right)}^{\prime }\#{\bar{y}}^{sr}+{\left({\bar{v}}^{r}B^{rs}\right)}^{\prime }\#A^{sr}L^{rr}{\bar{y}}^{rr}+{\left({\bar{v}}^{t}B^{ts}\right)}^{\prime }\#{\bar{y}}^{sr}+{\left({\bar{v}}^{t}B^{ts}\right)}^{\prime }\#A^{sr}L^{rr}{\bar{y}}^{rr}$$

Then, the value added parts in lithium battery exports from economy s to economy r should be:

(Equation 9) $${\text{dva}}_{LBT}=\bar{\text{dva}}-\text{dva}=\left({\left({\bar{v}}^{s}B^{ss}\right)}^{\prime }\#{\bar{y}}^{sr}+{\left({\bar{v}}^{s}L^{ss}\right)}^{\prime }\#A^{sr}B^{rr}{\bar{y}}^{rr}+{\left({\bar{v}}^{s}L^{ss}\right)}^{\prime }\#A^{sr}B^{rt}{\bar{y}}^{tt}+{\left({\bar{v}}^{s}L^{ss}\right)}^{\prime }\#A^{sr}B^{rr}{\bar{y}}^{rt}+{\left({\bar{v}}^{s}L^{ss}\right)}^{\prime }\#A^{sr}B^{rt}{\bar{y}}^{tr}\right)-\left({\left(v^{s}B^{ss}\right)}^{\prime }\#y^{sr}+{\left(v^{s}L^{ss}\right)}^{\prime }\#A^{sr}B^{rr}y^{rr}+{\left(v^{s}L^{ss}\right)}^{\prime }\#A^{sr}B^{rt}y^{tt}+{\left(v^{s}L^{ss}\right)}^{\prime }\#A^{sr}B^{rr}y^{rt}+{\left(v^{s}L^{ss}\right)}^{\prime }\#A^{sr}B^{rt}y^{tr}\right)$$

(Equation 10) $${\text{rva}}_{LBT}=\bar{\text{rva}}-\text{rva}=\left({\left({\bar{v}}^{s}L^{ss}\right)}^{\prime }\#A^{sr}B^{rr}{\bar{y}}^{rs}+{\left({\bar{v}}^{s}L^{ss}\right)}^{\prime }\#A^{sr}B^{rt}{\bar{y}}^{ts}+{\left({\bar{v}}^{s}L^{ss}\right)}^{\prime }\#A^{sr}B^{rt}{\bar{y}}^{ss}\right)-\left({\left(v^{s}L^{ss}\right)}^{\prime }\#A^{sr}B^{rr}y^{rs}+{\left(v^{s}L^{ss}\right)}^{\prime }\#A^{sr}B^{rt}y^{ts}+{\left(v^{s}L^{ss}\right)}^{\prime }\#A^{sr}B^{rt}y^{ss}\right)$$

(Equation 11) $${\text{fva}}_{LBT}=\bar{\text{fva}}-\text{fva}=\left({\left({\bar{v}}^{r}B^{rs}\right)}^{\prime }\#{\bar{y}}^{sr}+{\left({\bar{v}}^{r}B^{rs}\right)}^{\prime }\#A^{sr}L^{rr}{\bar{y}}^{rr}+{\left({\bar{v}}^{t}B^{ts}\right)}^{\prime }\#{\bar{y}}^{sr}+{\left({\bar{v}}^{t}B^{ts}\right)}^{\prime }\#A^{sr}L^{rr}{\bar{y}}^{rr}\right)-\left({\left(v^{r}B^{rs}\right)}^{\prime }\#y^{sr}+{\left(v^{r}B^{rs}\right)}^{\prime }\#A^{sr}L^{rr}y^{rr}+{\left(v^{t}B^{ts}\right)}^{\prime }\#y^{sr}+{\left(v^{t}B^{ts}\right)}^{\prime }\#A^{sr}L^{rr}y^{rr}\right)$$

The components in ${\text{dva}}_{LBT}$, ${\text{rva}}_{LBT}$ and ${\text{fva}}_{LBT}$ can be divided by the initial source and final target into five parts:

(Equation 12) $${\text{va}}_{LBT}^{sr}=\left({\left({\bar{v}}^{s}B^{ss}\right)}^{\prime }\#{\bar{y}}^{sr}-{\left(v^{s}B^{ss}\right)}^{\prime }\#y^{sr}\right)+\left({\left({\bar{v}}^{s}L^{ss}\right)}^{\prime }\#A^{sr}B^{rr}{\bar{y}}^{rr}-{\left(v^{s}L^{ss}\right)}^{\prime }\#A^{sr}B^{rr}y^{rr}\right)+\left({\left({\bar{v}}^{s}L^{ss}\right)}^{\prime }\#A^{sr}B^{rt}{\bar{y}}^{tr}-{\left(v^{s}L^{ss}\right)}^{\prime }\#A^{sr}B^{rt}y^{tr}\right)$$

(Equation 13) $${\text{va}}_{LBT}^{ss}=\left({\left({\bar{v}}^{s}L^{ss}\right)}^{\prime }\#A^{sr}B^{rr}{\bar{y}}^{rs}-{\left(v^{s}L^{ss}\right)}^{\prime }\#A^{sr}B^{rr}y^{rs}\right)+\left({\left({\bar{v}}^{s}L^{ss}\right)}^{\prime }\#A^{sr}B^{rt}{\bar{y}}^{ts}-{\left(v^{s}L^{ss}\right)}^{\prime }\#A^{sr}B^{rt}y^{ts}\right)+\left({\left({\bar{v}}^{s}L^{ss}\right)}^{\prime }\#A^{sr}B^{rt}{\bar{y}}^{ss}-{\left(v^{s}L^{ss}\right)}^{\prime }\#A^{sr}B^{rt}y^{ss}\right)$$

(Equation 14) $${\text{va}}_{LBT}^{st}=\left({\left({\bar{v}}^{s}L^{ss}\right)}^{\prime }\#A^{sr}B^{rt}{\bar{y}}^{tt}-{\left(v^{s}L^{ss}\right)}^{\prime }\#A^{sr}B^{rt}y^{tt}\right)+\left({\left({\bar{v}}^{s}L^{ss}\right)}^{\prime }\#A^{sr}B^{rr}{\bar{y}}^{rt}-{\left(v^{s}L^{ss}\right)}^{\prime }\#A^{sr}B^{rr}y^{rt}\right)$$

(Equation 15) $${\text{va}}_{LBT}^{rr}=\left({\left({\bar{v}}^{r}B^{rs}\right)}^{\prime }\#{\bar{y}}^{sr}-{\left(v^{r}B^{rs}\right)}^{\prime }\#y^{sr}\right)+\left({\left({\bar{v}}^{r}B^{rs}\right)}^{\prime }\#A^{sr}L^{rr}{\bar{y}}^{rr}-{\left(v^{r}B^{rs}\right)}^{\prime }\#A^{sr}L^{rr}y^{rr}\right)$$

(Equation 16) $${\text{va}}_{LBT}^{tr}=\left({\left({\bar{v}}^{t}B^{ts}\right)}^{\prime }\#{\bar{y}}^{sr}-{\left(v^{t}B^{ts}\right)}^{\prime }\#y^{sr}\right)+\left({\left({\bar{v}}^{t}B^{ts}\right)}^{\prime }\#A^{sr}L^{rr}{\bar{y}}^{rr}-{\left(v^{t}B^{ts}\right)}^{\prime }\#A^{sr}L^{rr}y^{rr}\right)$$

${\text{va}}_{LBT}^{sr}$ is the vaule added driven by lithium battery trade from sectors in economy s to economy r (similar with ${\text{va}}_{LBT}^{ss}$, ${\text{va}}_{LBT}^{st}$, ${\text{va}}_{LBT}^{rr}$ and ${\text{va}}_{LBT}^{tr}$). ${va}_{LBT,i}^{sr}$ denotes the vaule added driven by lithium battery trade from sector i in economy s to economy r. Aggregating the source of vaule added from sectors to economies, we obtain:

(Equation 17) $${vc}_{LBT}^{sr}=\sum_{i}{va}_{LBT,i}^{sr}$$

${vc}_{LBT}^{sr}$ denotes the vaule added driven by lithium battery trade from economy s to economy r (similar with ${vc}_{LBT}^{ss}$, ${vc}_{LBT}^{st}$, ${vc}_{LBT}^{rr}$ and ${vc}_{LBT}^{tr}$).

By do so, we decompose the lithium battery trade from economy s to economy r into the value added flows among economy s, r and t.

#### Lithium-battery-trade-driven embodied carbon emission accounting

In the input-output analysis, embodied carbon emission coefficient vector of all sectors is usually calculated as:

(Equation 18) $$\varepsilon =d{\left(I-A\right)}^{-1}$$

d is the vector of direct carbon emission intensity. Each value of d is calculated as $d_{i}^{s}=\frac{{ce}_{i}^{s}}{x_{i}^{s}}$. ${ce}_{i}^{s}$ is the direct carbon emissions of sector i in economy s. $x_{i}^{s}$ is total output of sector i in economy s. I is the identity matrix. A is the direct consumption matrix.

By multiplying embodied carbon emissions coefficient by final consumption, the carbon emissions embodied in the lithium battery trade can be calculated as:

(Equation 19) $${ec}_{LBT}^{sr}=\varepsilon_{\phi }^{s}t^{sr}$$

${ec}_{LBT}^{sr}$ denotes the carbon emissions embodied in the lithium battery trade from economy s to economy r. ϕ denotes the sector which produce lithium battery. $\varepsilon_{\phi }^{s}$ is the embodied carbon emission coefficient of sector ϕ in economy s. $t^{sr}$ is the trade value of the lithium battery exported to economy r from economy s.

#### LBTVEFN construction and analysis

##### Network construction

We define economies as nodes and define value added flows between economies as edges. Then we obtain the LBTVAFN. The weight of value added flow from economy A to economy B denotes the value added in the exports related to lithium batteries from economy A to economy B, which measures the economic incomes for economy A and the economic costs for economy B. We define economies as nodes and define embodied carbon emission flows between economies as edges. Then we obtain the LBTCEFN. The weight of embodied carbon emission flow from economy A to economy B denotes the carbon emissions embodied in the exports related to lithium batteries from economy A to economy B, which is the carbon emissions driven by lithium battery exports of economy A and the carbon emissions driven by lithium battery imports of economy B. Due to the shared nodes in LBTVAFN and LBTCEFN, a two-layer network LBTVEFN is constructed (Figure S2).

##### Network analysis

###### Overall network characteristics

Here, we choose four network indexes to measure the overall structure characteristics of LBTVAFN and LBTCEFN, including the average degree, the average weighted degree, the average path length and the average clustering coefficient. The average degree usually measures the average exchange partners of economies in value added and embodied carbon emission transfers. The average weighted degree usually measures the average transfer volumes of value added and embodied carbon emissions. The average path length and the average clustering coefficient are usually used to measure the density of networks.

The average degree can be calculated as follows:

(Equation 20) $$K=\frac{\sum_{i=1}^{n}\sum_{j=1}^{n}A_{i,j}}{n}$$

where n is the number of economies. A is the adjacent matrix of the network. If there is an edge between economy i and economy j, $A_{i,j}=1$; otherwise, $A_{i,j}=0$.

The average weighted degree can be calculated as follows:

(Equation 21) $$W=\frac{\sum_{i=1}^{n}\sum_{j=1}^{n}w_{i,j}}{n}$$

where n is the number of economies. $w_{i,j}$ is the volume of value added flows or embodied carbon emission flows between economy i and economy j.

The average shortest path length is usually calculated as follows:

(Equation 22) $$L=\frac{1}{n-1}\sum_{i\neq j}^{n}l_{i,j}$$

where n is the number of economies. $l_{i,j}$ denotes the shortest path lengths between economy i and economy j.

The average clustering coefficient can be calculated as follows:

(Equation 23) $$C=\frac{1}{n}\sum_{i=1}^{n}\frac{2e_{i}}{k_{i}\left(k_{i}-1\right)}$$

where n is the number of economies. $k_{i}$ is the degree of economy i. $k_{i}\left(k_{i}-1\right)/2$ represents the theoretical number of edges between economy i and its neighbors. $e_{i}$ is the actual number of edges between economy i and its neighbors.

Furthermore, we choose the (weighted) degree correlation coefficient and cosine similarity index to measure the structure correlations between the LBTVAFN and the LBTCEFN.

In research on multiplex networks, scholars usually choose the Person correlation coefficient to measure the interlayer degree correlation coefficient, which is shown as:

(Equation 24) $$\gamma_{\alpha ,\beta }=\frac{\left\langle k_{i}^{\left[\alpha \right]}k_{i}^{\left[\beta \right]}\right\rangle -\left\langle k_{i}^{\left[\alpha \right]}\right\rangle \left\langle k_{i}^{\left[\beta \right]}\right\rangle }{\sigma_{k^{\left[\alpha \right]}}\sigma_{k^{\left[\beta \right]}}}$$

where α,β denote the LBTVAFN layer and the LBTCEFN layer respectively. $k_{i}^{\left[\alpha \right]}$ denotes the (weighted) in-degree (out-degree) of economy i in the LBTVAFN. $\left\langle k_{i}^{\left[\beta \right]}\right\rangle$ denotes the average (weighted) in-degree (out-degree) of all economies in the LBTCEFN. $\sigma_{k^{\left[\alpha \right]}}$ is the standard deviation of the degree of all economies in the LBTVAFN. $\sigma_{k^{\left[\beta \right]}}$ is the standard deviation of the degree of all economies in the LBTCEFN.

The cosine similarity index can be calculated as follows:

(Equation 25) $$\phi \left(x,y\right)=\frac{x\cdot y}{\left\|x\right\|\cdot \left\|y\right\|}$$

where x and y are two ordered vectors of pairwise economies that interact with each other in the LBTVAFN and the LBTCEFN respectively.

###### Roles of economies

Here, we choose four network indexes to measure the roles of economies in the LBTVAFN and the LBTCEFN, including the weighted out-degree, the weighted in-degree, the betweenness centrality and the clustering coefficient. The weighted out-degree of one economy measures the value added or embodied carbon emissions in the exports related to lithium batteries, which reflects its economic incomes or carbon emissions driven by lithium battery exports. The weighted in-degree of one economy measures the value added or embodied carbon emissions in the imports related to lithium batteries, which reflects its economic costs or carbon emissions driven by lithium battery imports. The betweenness centrality usually measures the media capability of economies in the network. Economies with high betweenness centrality usually means its media capability in the value added or embodied carbon emission flows. The clustering coefficient measures the probability that the adjacent economies of an economy are connected in the network. Economies with high clustering coefficient usually means there are close value added or embodied carbon emission flows between the its neighbors.

The weighted in-degree and the weighted out-degree are calculated as follows:

(Equation 26) $${SI}_{i}=\sum_{j\epsilon N_{i}}w_{j,i}$$

(Equation 27) $${SO}_{i}=\sum_{j\epsilon N_{i}}w_{i,j}$$

where $N_{i}$ is the set of economies connected to economy i. $w_{j,i}\left(w_{i,j}\right)$ is the weight of the edge from economy j (i) to economy i (j).

The betweenness centrality is calculated as follows:

(Equation 28) $${BC}_{i}=\frac{\sum_{j}^{n}\;\sum_{k}^{n}g_{jk}\left(i\right)/g_{jk}}{\left(n^{2}-3n+2\right)/2},j\neq k\neq i,j<k$$

where $g_{jk}\left(i\right)$ denotes the number of shortest paths from economy j to economy k that pass through economy i. $g_{jk}$ denotes the number of the shortest paths from economy j to economy k.

The formula for calculating the clustering coefficient is:

(Equation 29) $$C_{i}^{w}=\frac{1}{s_{i}\left(k_{i}-1\right)}\sum_{j,h}\frac{w_{i,j}+w_{i,h}}{2}a_{i,j}a_{i,h}a_{j,h}$$

where $s_{i}$ is the strength of economy i. $a_{i,j}$ represents the element of the adjacency matrix. $k_{i}$ denotes the degree of economy i. $w_{i,j}$ is the weight of the edge from economy i to economy j.

### Quantification and statistical analysis

Quantitative analysis was conducted using Excel, Matlab, R script and Origin and the results were reflected in Figure 1, Figure 2, Figure 3, Figure 4, Figure 5, Figure 6, Figure 7.
