# Supplementary material for: Global value added and embodied carbon emission transfers driven by lithium battery trade
Source: iScience. 2025 Mar 30;28(5):112320. doi: 10.1016/j.isci.2025.112320 (PMC12033944; doi:10.1016/j.isci.2025.112320)
Supplement: Document S1. Figures S1 and S2 and Tables S1 and S2 [file mmc1.pdf]

iScience, Volume *28*

Supplemental information

Global value added and embodied carbon emission transfers driven by lithium battery trade

Meihui Jiang, Xiaoqing Hao, Bingyan Li, Wenlin Cai, and Yanfang Zhang

Supplementary Information

Supplementary Figures:

<table>
<tr><td colspan="3" rowspan="3">Output / Output</td><td colspan="6">Intermediate output</td><td colspan="3">Final consumption</td><td rowspan="3">Total ouput</td></tr>
<tr><td colspan="2">Economy r</td><td colspan="2">Economy s</td><td colspan="2">Economy t</td><td rowspan="2">Economy r</td><td rowspan="2">Economy s</td><td rowspan="2">Economy t</td></tr>
<tr><td>S1</td><td>S2</td><td>S1</td><td>S2</td><td>S1</td><td>S2</td></tr>
<tr><td rowspan="6">Intermediate input</td><td rowspan="2">Economy r</td><td>S1</td><td colspan="2" rowspan="2">$\mathbf{Z}^{rr}$</td><td colspan="2" rowspan="2">$\mathbf{Z}^{rs}$</td><td colspan="2" rowspan="2">$\mathbf{Z}^{rt}$</td><td rowspan="2">$\mathbf{y}^{rr}$</td><td rowspan="2">$\mathbf{y}^{rs}$</td><td rowspan="2">$\mathbf{y}^{rt}$</td><td rowspan="2">$\mathbf{x}^{r}$</td></tr>
<tr><td>S2</td></tr>
<tr><td rowspan="2">Economy s</td><td>S1</td><td colspan="2" rowspan="2">$\mathbf{Z}^{sr}$</td><td colspan="2" rowspan="2">$\mathbf{Z}^{ss}$</td><td colspan="2" rowspan="2">$\mathbf{Z}^{st}$</td><td rowspan="2">$\mathbf{y}^{sr}$</td><td rowspan="2">$\mathbf{y}^{ss}$</td><td rowspan="2">$\mathbf{y}^{st}$</td><td rowspan="2">$\mathbf{x}^{s}$</td></tr>
<tr><td>S2</td></tr>
<tr><td rowspan="2">Economy t</td><td>S1</td><td colspan="2" rowspan="2">$\mathbf{Z}^{tr}$</td><td colspan="2" rowspan="2">$\mathbf{Z}^{ts}$</td><td colspan="2" rowspan="2">$\mathbf{Z}^{tt}$</td><td rowspan="2">$\mathbf{y}^{tr}$</td><td rowspan="2">$\mathbf{y}^{ts}$</td><td rowspan="2">$\mathbf{y}^{tt}$</td><td rowspan="2">$\mathbf{x}^{t}$</td></tr>
<tr><td>S2</td></tr>
<tr><td colspan="3">Value added</td><td colspan="2">$\mathbf{va}^{r}$</td><td colspan="2">$\mathbf{va}^{s}$</td><td colspan="2">$\mathbf{va}^{t}$</td><td colspan="4"></td></tr>
<tr><td colspan="3">Total input</td><td colspan="2">$(\mathbf{x}^{r})'$</td><td colspan="2">$(\mathbf{x}^{s})'$</td><td colspan="2">$(\mathbf{x}^{t})'$</td><td colspan="4"></td></tr>
</table>

Figure S1. The structure of a multi-regional input-output table. Related to STAR Method.

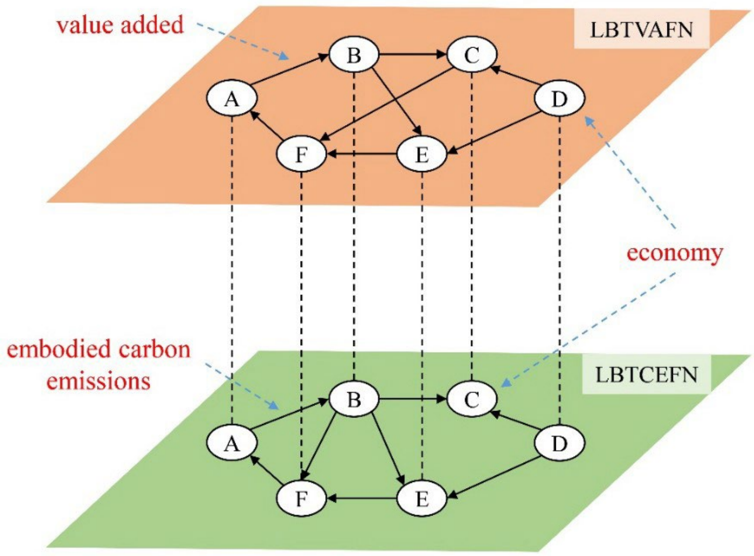

Figure S2. The schematic diagram of LBTVEFN. Related to STAR Method.

Note: Node with letters denotes economy. Solid arrow between nodes denotes flow of value added and embodied carbon emissions between economies. Dotted line between nodes means two nodes are one economy.

Supplementary Tables:

Table S1. The trade value of lithium batteries of the top 10 economies in 2021

No.	Economies	Export value (million euro)	Economies	Import value (million euro)
1	China	36771.9	Germany	18622.6
2	Poland	12890.8	USA	16101.8
3	South Korea	8568.0	South Korea	5549.6
4	Germany	8314.1	China	4016.0
5	Ireland	6045.7	Mexico	3387.6
6	Japan	5188.7	Greece	3091.9
7	USA	2891.8	Czech Republic	2789.1
8	Czech Republic	1238.8	Netherlands	2717.3
9	Netherlands	1149.4	Spain	2711.9
10	Mexico	656.9	Slovakia	2660.5

Data source: the results in Table S1 are obtained from the trade data of lithium batteries in UN COMTRADE database.

Table S2. Economy list

No.	Name	No.	Name
1	Austria	23	Portugal
2	Belgium	24	Romania
3	Bulgaria	25	Sweden
4	Cyprus	26	Slovenia
5	Czech Republic	27	Slovakia
6	Germany	28	The UK
7	Denmark	29	The USA
8	Estonia	30	Japan
9	Spain	31	China
10	France	32	Canada
11	Greece	33	South Korea
12	Croatia	34	Brazil
13	Hungary	35	India
14	Ireland	36	Mexico
15	Italy	37	Russia
16	Lithuania	38	Australia
17	Luxembourg	39	Switzerland
18	Finland	40	Turkey
19	Latvia	41	Norway
20	Malta	42	Indonesia
21	Netherlands	43	South Africa
22	Poland		
